# The learner’s perspective in GP teaching practices with multi-level learners: a qualitative study

**DOI:** 10.1186/1472-6920-14-55

**Published:** 2014-03-19

**Authors:** Jennifer S Thomson, Katrina Anderson, Emily Haesler, Amanda Barnard, Nicholas Glasgow

**Affiliations:** 1 Academic Unit of General Practice, Medical School, College of Medicine, Biology & Environment, Australian National University, Acton, ACT, Australia; 2 Rural Clinical School, Medical School, College of Medicine, Biology & Environment, Australian National University, Acton, ACT, Australia; 3 Medical School, College of Medicine, Biology & Environment, Australian National University, Acton, ACT, Australia

**Keywords:** Postgraduate training, Workplace based learning, General Practice Education, Teaching innovation, Vertical integration of GP education, Multi-level learning, Learning culture

## Abstract

**Background:**

Medical students, junior hospital doctors on rotation and general practice (GP) registrars are undertaking their training in clinical general practices in increasing numbers in Australia. Some practices have four levels of learner. This study aimed to explore how multi-level teaching (also called vertical integration of GP education and training) is occurring in clinical general practice and the impact of such teaching on the learner.

**Methods:**

A qualitative research methodology was used with face-to-face, semi-structured interviews of medical students, junior hospital doctors, GP registrars and GP teachers in eight training practices in the region that taught all levels of learners. Interviews were audio-recorded and transcribed. Qualitative analysis was conducted using thematic analysis techniques aided by the use of the software package N-Vivo 9. Primary themes were identified and categorised by the co-investigators.

**Results:**

52 interviews were completed and analysed. Themes were identified relating to both the practice learning environment and teaching methods used.

A practice environment where there is a strong teaching culture, enjoyment of learning, and flexible learning methods, as well as learning spaces and organised teaching arrangements, all contribute to positive learning from a learners’ perspective.

Learners identified a number of innovative teaching methods and viewed them as positive. These included multi-level learner group tutorials in the practice, being taught by a team of teachers, including GP registrars and other health professionals, and access to a supernumerary GP supervisor (also termed “GP consultant teacher”). Other teaching methods that were viewed positively were parallel consulting, informal learning and rural hospital context integrated learning.

**Conclusions:**

Vertical integration of GP education and training generally impacted positively on all levels of learner. This research has provided further evidence about the learning culture, structures and teaching processes that have a positive impact on learners in the clinical general practice setting where there are multiple levels of learners. It has also identified some innovative teaching methods that will need further examination. The findings reinforce the importance of the environment for learning and learner centred approaches and will be important for training organisations developing vertically integrated practices and in their training of GP teachers.

## Background

The demand for education and training opportunities in clinical general practice is increasing worldwide [1]. In Australia, the number of medical students and junior doctors has doubled and general practice (GP) registrars have trebled in the last ten years [2,3]. With this increasing demand, some teaching general practices now accommodate medical students, junior doctors on rotation from a hospital as part of the Prevocational General Practice Placement Program (PGPPP) [4,5] and GP registrars [6]. Teaching learners at different levels of the learning continuum in the clinical setting is termed ‘vertical integration of GP education and training’ (VI) [7].

An individual, learner-centred approach has been accepted as essential in any medical education curriculum to promote deep learning. Such approaches include self-directed learning and adult learning principles [8]. Other elements that have been identified as essential for effective medical education include planned teaching, structured experiences, role models and supportive learning environments [9].

The learning environment is now recognised as important in enabling effective education [10]. Learning in general practice takes place in a complex clinical environment with a small number of clinicians and ancillary staff. Smith et al. identified that the learning climate in general practice needs to be better understood and managed [11].

While multi-level learning has been delivered in the hospital environment for many years, understanding of its impact in the clinical general practice setting is limited, particularly the impact on the learner. A number of papers have described some of the impacts of VI. Dick et al. [12] proposed that VI teaching and learning had the potential to increase efficiency, improve learning experiences and improve teaching capacity. Studies have also identified enablers of and barriers to VI teaching at the practice level, including teaching capacity, time and availability of space and financial viability [13,14]. Van de Mortel et al. [15] identified that shared learning was promoted by enabling factors such as small group facilitation, reinforcing factors such as targeted funding and predisposing factors such as participant attributes. Brumpton et al. [16] have identified that different learning preferences of GP registrars and medical students should be considered in VI.

Opportunity to learn from and alongside medical students, registrars and junior doctors has also been identified as a motivator for GP participation in teaching [17]. An assessment of a VI model delivered in South Australia suggested that a VI training model was associated with an increase in workplace numbers through the attraction to the region of both experienced and training practitioners [18]. Morrison et al. [19] suggested that multi-level learning impacts the entire general practice, including patients and non-medical staff. In a recent evaluation of integrated educational workshops for rural stream medical students and GP registrars, participants responded favourably to the joint educational experience in a large workshop setting [20].

Training practices have developed a range of VI models alongside expansion of their teaching capacity. Differing learner needs and supervisor delivery preferences have led to variability in the emergence of VI models in the GP setting [21,22].

The impact on learners is a key outcome for any education delivery. A UK study [11] identified that issues relating to the practice, such as team relationships; flexibility of teaching; and the knowledge, skill and attitudes of the GP trainer are important to the learner. The experiences of, and benefits for students, junior doctors and registrars learning jointly in a general practice have had minimal focus in the literature.

The aim of this research was to describe how VI is delivered in practices and to identify the elements associated with a positive impact on learning. The research findings will contribute to the ongoing development of a sustainable learner-centred education delivery approach, as well as provide insight into elements of dynamic team based general practice learning environments that support learning.

## Method

This research explored VI in clinical general practices and the impact on learners of the VI experience in that context. As our aim was to gain in-depth insight into trainees’ learning experiences, we used a qualitative research methodology using a phenomenological approach [23]. The phenomenon under scrutiny in this research was the learners’ VI experience in clinical general practice. A phenomenological approach enabled us to gain a broad understanding of the practical implementation of VI models and to explore the learners’ unique perspectives of the effectiveness of these models in order to locate and interpret their shared experiences. We utilised face-to-face in-depth interviews to gain insight into the learners’ lived experiences of teaching and learning.

The primary investigators had key involvement in the promotion of VI teaching at the university, and have personal experience as GP supervisors in general practices delivering VI initiatives. As GPs involved in teaching in the region, they had an intrinsic understanding of many of the factors the participants face in their clinical practice and teaching roles. The interviewers were not directly involved in any of the region’s teaching programs.

### Ethics approval

Ethics approval was obtained through the Australian National University Human Research Ethics Committee (protocol 2011/415). All participants received written and verbal information about the research project before signing a consent form to participate.

### Selection of teaching practices

The research was conducted in one of seventeen GP training regions in Australia. A purposive sampling technique was used to identify teaching practices within the region to participate in the study. The sampling framework for selection of general practices included representation of:

• Different VI models (e.g. a variety in administrative or teaching strategies);

• Urban and rural geographic locations; and

• Combinations of levels of learners (i.e. medical student, junior doctor, GP registrar, GP supervisor).

Because the VI model of learning focuses on learning at all levels, incorporating the concept of life-long learning, GP teachers were considered to be learners in this context and were therefore included in the study.

All vertically integrated teaching practices in the region with experience in having all four levels of learner were selected for inclusion. This represented eight out of a total of 52 teaching practices in the region. Practice owners were approached with an introductory letter outlining the opportunity to participate and explaining the potential benefits for the teaching within their practice.

### Selection of individual participants within the practices

After the practice owner had agreed to the practice’s participation, individual learners currently or recently engaged in VI teaching at the practice were provided with information about the study and invited to participate. This included:

• Medical students on short and long term placements;

• GP registrars;

• PGPPP junior doctors and

• GP supervisors.

### Interviews

Participants engaged in face-to-face, semi-structured interviews. Two primary investigators (JT and EH) conducted the interviews over a 9-month period between October 2011 and June 2012 at locations convenient to the interviewees (often within the practice in which they were currently training). The interviewers had no direct relationship with any of the participants although in some cases were known to them. Prior to the interview, participants completed a survey of demographic details. Interviews were conducted using a set of prompt questions (see the List of Prompt interview questions) that explored the experiences of learners in the VI model and identified components associated with both positive and negative learning experiences. Interviews were audio-recorded with the consent of the interviewee and identified using non-descript codes to which only the project manager had access.

### List of prompt interview questions

1. How has being in this practice enabled your learning?

2. Can you describe how VI learning happens in your practice?

3. Are there any key benefits that you attained from participation in the VI learning in the practice?

4. Are there any problems with participation in VI learning in the practice?

5. Describe a successful VI learning experience that you had in your practice. What are the features that have made it successful?

6. Describe an experience that was not so successful. What were the features that made it not so successful?

7. Overall, were you satisfied with the VI learning experience in the practice? Why/why not?

8. How was your relationship with other learners or teachers affected by VI learning?

9. Can you identify who has had the biggest impact on your learning in the practice and why?

10. How does the level of the teacher influence your learning *e.g. GP Registrar vs. GP Supervisor*?

11. Do you enjoy learning situations where there are learners with different levels of experience? Can you elaborate (why, what, how, when?)

12. Describe how your confidence as a doctor/student was affected by VI learning.

13. Describe how your competence as a doctor/student was affected by VI learning.

### Data analysis

Qualitative analysis of the interview transcripts was conducted using a thematic analysis approach aided by the use of the software package N-Vivo 9 [24]. One of the researchers with considerable experience in qualitative data analysis (EH) read all the transcripts as the interviews were being conducted and identified initial meaning units using open coding. In the second stage of analysis, two of the researchers (JT and EH) read the coded extracts from all interviews and together they reduced the coding set into 28 primary themes through removal of duplications and grouping of similar or overlapping categories.

There was strong agreement between the researchers as to the identified codes and their relevance to the topic of enquiry. Following construction of the coding set, the transcripts were re-read (EH) to ensure that all relevant text fragments for each code were identified. Ongoing discussions between the researchers during analysis of the coding identified relationships between the codes and after discussion between four of the researchers (JT, EH, KA, AB), the themes were organized within two higher order codes, with one reflecting learning environment and culture and the other reflecting teaching methods and supervision arrangements, as these appeared to be the two most commonly reported aspects of GP practice based clinical teaching being described by interviewees. The 28 primary codes were then condensed into 14 subthemes and grouped into the two higher order codes as outlined in Table 1. Ongoing discussion between all the research investigators of the emerging data provided additional focus for interviews as they progressed, allowing for further confirmation and exploration of identified themes.

**Table 1:** Factors that facilitated learning

**Practice learning environment**	**Teaching methods**
Enjoyment	Group tutorials
Teaching ethos and culture	Informal teaching
Flexibility	Parallel consulting
Administrative support	GP registrars as teachers
Space	Rural hospital experience
Remuneration	Supernumerary GP supervisor
	The teaching team
	Interdisciplinary teaching

## Results

### Characteristics of participants

Eight teaching practices in the region with experience in delivering VI models of learning for medical students, PGPPP junior doctors and GP registrars were recruited to participate. Four were rural practices and four were urban. Seven of these practices had all four levels of learner at the practice (i.e. medical students, PGPPP junior doctor, GP registrar, GP supervisor). The other practice was awaiting allocation of a PGPPP junior doctor. Seven of the practices were owned by the principal GP supervisors and one was a community-controlled organisation.

Fifty-two individuals participated in interviews regarding their experience as a learner in a VI model of teaching. Table 2 outlines the level of learner and the location of the practice in which they engaged in VI learning.

**Table 2:** Interview participant characteristics

	**Urban practice**	**Rural practice**	**Total**
Medical students	1	8	9
PGPPP (junior doctors)	11	2	13
GP registrars	5	10	15
GP supervisors	7	8	15
	**24**	**28**	**52**

Medical students were primarily located in rural practices and enrolled in a long-term rural stream placement. Students had generally spent six weeks training in the general practice prior to participating in an interview. Difficulty was faced in recruiting medical students due to university holiday periods, study and exam commitments during the study time frame.

Doctors enrolled in the PGPPP training program were in their intern year in urban practices and second post-graduate year in rural practices. They had spent between four and twelve weeks in the general practice at the time of their interview.

GP registrar participants were primarily in their second and third (or more) year of registrar training. They had been in the teaching general practice enrolled in the study for between one and three years, although the majority had spent about one year in the specific practice.

The vast majority of GP supervisor participants had been located in the general practice enrolled in the study for over 10 years. About 30% of the GP supervisors had a higher qualification in education as well as an academic title.

### Interview results

The learners described features of integrated practices and practical models of VI teaching that they viewed as positive. They are described in detail below, with indicative quotes.

#### Practice learning environment

Many of the learners in these integrated practices identified particular features of the teaching practice as prerequisites for successful learning. These included both cultural factors and organisational factors. Interviewees identified GP supervisors who were also practice owners (practice principals) as clear champions in creating a positive learning environment.

##### Cultural factors

All levels of learner identified cultural factors, such as a strong teaching ethos with enjoyable and flexible, learner-centered teaching as core elements in creating a positive learning environment. Many learners identified the practices as having a strong teaching ethos and culture, generally driven by the senior GP supervisors.

“A lot of the senior GPs are involved with the medical school and it’s (the practice)… just got that culture to it I suppose, teaching culture.” (Registrar 16)

Practice principals expressed their decision to make teaching one of the unique features of their practice and as such, were happy to expand their teaching effort to include a wide range of learners.

Enjoyment of teaching and motivation to engage in ongoing, lifelong learning was considered important in sustaining the engagement of learners and also in preventing burnout and teacher fatigue. Interviewees of all learning levels were able to identify the culture of enjoyment and motivation in the practice principals, and sometimes in other learners of a higher level.

GP supervisors expressed a need to continually re-evaluate their teaching structures and delivery to meet the needs of learners. Learners reported that GP supervisors in these practices were clearly responsive to their needs, flexible regarding the type of learning opportunities being provided, and able to deliver teaching in innovative ways.

“…*because with the senior GPs, because they actually take a whole lot of interest in our learning,… they’re a bit more flexible.” (Student 3)*

##### Organisational factors

Learners identified organisational factors, such as teaching organisation, space and financial arrangements as important in creating an environment for effective learning. Learners indicated that in order to work well, teaching time and supervision arrangements needed to be scheduled and organised.

“There was time to teach and to learn. They booked in fewer patients when they were supervising interns and that made a big difference.” (PGPPP 11)

The physical learning environment was identified by learners as fundamental to an enhanced VI learning experience. Junior learners mentioned consulting rooms set aside for learners, a good-sized tearoom and a meeting room as essential for both informal and formal learning.

Several respondents discussed how the “closed door” patient consultation environment of general practice was a different experience from the hospital context. This gave medical students and junior doctors a sense of autonomy, of seeing their “own” patients, having to make clinical decisions and write clinical records before presenting their patient to their supervisor.

“I think it makes you feel like more of a doctor than sometimes in hospital where you are just running around with a paper trail doing chores for other people. So I think you feel more like a real doctor having your own room, seeing your own patients.” (PGPPP 11)

There was also the risk of isolation and developing bad habits if unobserved with the patient.

“..You’re in your own room all day seeing patients so you do run the risk of establishing bad habits..” (Registrar 16)

Some supervisors highlighted that integrated learning models improved the financial viability of teaching in the practice while others indicated they earned less from consulting when they took on a teaching load. The supervisors considered that financial decisions may influence the teaching participation of some GPs.

#### Teaching methods

The learners identified methods of teaching in integrated practices that enhanced their learning. The VI teaching techniques most commonly identified as providing positive learning experiences are described below. In some cases learners also identified problems with teaching methods.

##### Multi-level learner tutorials

Regularly scheduled tutorials (usually weekly) conducted in the practice appeared to be the most commonly used method of integrated teaching. Practice-based multi-level learning experiences usually included all four levels of learner: GP supervisor, GP registrar, junior doctor and medical student. In most cases all participants were given the opportunity to present topics or cases and lead discussions. Learners identified both benefits and problems with such tutorials. Benefits of multi-level tutorials for learners of all levels included:

“…it makes you think OK well you don’t have to know everything by the time you’ve finished, you’re still going to be in a supported learning environment for quite a number of years to come and that’s encouraging to see.” (Student 4)

• Creation of an equal partnership in learning in which all members of the group contributed to presentations;

• A supportive learning environment in which members of the group were able to problem solve together;

• Provision of future learning direction for junior learners as they saw first-hand discussions regarding the clinical challenges of general practice. Learners appreciated the importance of a sound knowledge base to apply to case-based problem solving;

• Provision of opportunity for more experienced clinicians to revise or update.

Some of the problems and challenges with multi-level tutorials identified by learners included:

“..some of the disadvantages are that if you are doing one topic or something it might suit one person much more than the others…if you ask people what they want to do the most forceful personality is probably going to come out and say yes I want to do this, or no I’m not interested in that.” (Supervisor 1)

• Meeting the varying learning needs of a group with different levels of clinical experience, knowledge and skills base;

• Managing different personalities in a small group setting;

• Exclusion of some learners from multi-level teaching sessions due to clashes with the educational organisation’s teaching requirements (e.g. urban PGPPP junior doctors were sometimes unable to attend as they have off-site tutorials provided at the Medical School).

##### Informal teaching

All levels of learner discussed informal learning interactions between different levels of learners as being of benefit to their learning experience. The practice tearoom was often described as the site for such interactions, and “corridor learning” was also seen as important. These interactions were usually spontaneous and often related to specific patient–based discussion, but also included discussion of broad medical issues and career planning.

“I always thought that tearooms are incredibly important places for any institution, and really functional general practices always have quite vibrant tearooms. And so it’s really important I think that the interns and the registrars are actually able to communicate with the others in the tearoom, and a lot of learning occurs.” (Supervisor 16)

##### Parallel consulting

A common observation among junior learners was various benefits offered by a specific VI teaching model, parallel consulting^1^, which was seen to offer the learner:

“I’ve learned much more in the GP practices by doing things hands on, seeing patients on my own and after that consulting with the doctors themselves and then having the debriefing session.” (Student 2)

• Autonomy in patient care;

• Expert supervision for all patient encounters;

• Increased opportunity to learn about patient management and

• One-on-one teaching.

Most GP supervisors also considered this to be a successful means of teaching. They reported that patient throughput is maintained, learners appear to benefit and patients also seem to appreciate this form of consulting.

However, some GP supervisors found parallel consulting to be time consuming, particularly if it affected patient flows. GP registrars mentioned that patients can be kept waiting if the supervisor is not readily available to sign off on consultations.

##### GP registrars as teachers

Medical students expressed overwhelmingly positive experiences learning from GP registrars. GP registrars were considered to be providing learning experiences that are as good as, but different from, those provided by GP supervisors. They indicated that registrars provide teaching that meets the student’s learning needs in terms of content and level of complexity because registrars have their own recent experience as a medical student, and are often still using a more methodical approach to their consultations. Junior learners viewed the GP registrars as easily approachable fellow learners who provided guidance and role modeling. However, GP registrars were not always able to provide targeted teaching related to learning needs, variety in learning experiences or flexibility in teaching arrangements, as were the GP supervisors.

“I feel there’s a greater degree of camaraderie with someone who’s more junior than someone who is much more senior.” (Student 7)

“The supervisors are a bit more capable it seems of identifying, from what I’ve said, what’s been missed and just honing in on those areas. Whereas a registrar seems to run their own thing.” (Student 4)

GP registrars indicated that their engagement in teaching was primarily determined by the teaching practice’s model rather than the registrar’s desire to be involved in teaching (e.g. the practice implemented a teaching timetable in which registrars were required to participate). It was also reported that the type of teaching in which the GP registrar engaged was directed by the teaching practice’s preferences.

##### Rural hospital experience

For those undertaking training in a rural general practice, the rural hospital was often mentioned as an important component of the vertically integrated clinical experience. Learners valued the integrated learning that is occurring in these contexts, with ward rounds involving all levels of learners and GP registrars often supervising medical students and PGPPP junior doctors in clinical situations.

“Most of the doctors at that practice work at the hospital as well sometimes, so there’s sort of a bit more opportunity for that [case conferences] there.” (Student 1)

Integrated learning activities taking place in the rural hospital context tended to mirror those in large teaching hospitals. These included multi-level interdisciplinary ward rounds, case conferences and registrar responsibility for supervision and teaching of junior learners. All levels of learner saw the multi-level learning that took place at the rural hospitals as useful to their learning.

##### Consultant GP teacher – supernumerary GP supervisor

Three of the seven practices indicated that they were trialling the concept of a GP supervisor who consults none of their own patients (or very limited number) throughout a session while they undertake responsibility for supervising all the learners in the practice. This supernumerary GP supervisor or “consultant GP teacher” undertakes parallel consulting with both a medical student and PGPPP junior doctor simultaneously, while also being available to assist the GP registrar if required. Different supervisors in two of the practices shared this role.

All levels of learner reported that this teaching model was very effective for the learner as there was ready access to a consultant GP teacher. However, practice principals were mixed in opinion about the financial cost involved in this supernumerary supervisor model.

##### The interdisciplinary teaching team

Learners enjoyed having exposure to a range of teachers in the practice. Teaching teams involved a combination of any of the GPs working in the practice in addition to those formally accredited as the GP supervisors. Some supervised learners at all levels, while others focused on a particular learner level. Supervisors saw that the team approach was important in sustaining the teaching effort, but also an opportunity for supervisors to share experiences and learn from each other.

“Whereas now, there’s probably more potential to have a, sort of a little bit of a team.” (Supervisor 18)

Learners described teaching teams that included GP registrars, practice manager, nurses, allied health or other clinical staff such as visiting specialists. The roles of each member of the team varied from supervising to delivering one-on-one tutorials, direct clinical teaching or presenting at group tutorials. The learners discussed how the team teaching approach gave them the opportunity to view different styles of consulting as well as to learn from clinicians with varying special interests.

“The good thing about this practice, it’s just got so many different specialties, so having a psychiatrist across the hallway was fantastic, having the nursing staff, having a dietician there that I could just grab and ask questions. I found that so rewarding and so interesting…” (PGPPP 7)

Learners at most of the integrated teaching practices described some level of interdisciplinary teaching and learning. Practice nurses were seen as valuable teachers, particularly for skills such as wound management, immunisation, health assessments and organisation of management plans. Learners mentioned that some practices also had medical specialists and allied health practitioners engaged in teaching within the practice context, and this evolving component of the learning experience was valued by the learner. A number of practices also included nursing students in their vertically integrated learning.

## Discussion

This study has identified a number of factors that enhance and support the learner in vertically integrated teaching practices with multi-level learners. The findings present some options for consideration by teaching general practices, teaching organisations and policy and funding bodies.

### Practice learning environment

Integrated practices that teach different levels of learners are becoming more numerous, and this research indicates that they provide a supportive and positive environment for learners. We identified three core underpinning factors as important platforms for successful multi-level learning in the general practice environment: a strong teaching culture and ethos; enjoyment of learning; and flexibility in teaching delivery. Our findings support those of Smith et al. in the UK [11] who identified that the learning environment impacts on learner satisfaction and attitude to learning. GP supervisors may play a major role in creating this positive learning environment.

GP supervisors owned the practices in this research, except for one community controlled service. Large practices owned by non-GPs did not participate in this form of integrated teaching in our region. The strategic commitment of general practice owners to teach within their businesses appears fundamental to allowing the multiple learners access to patients and hands-on learning. Commitment by these integrated teaching practices to teach, when there is no requirement to do so, must be valued, recognised and rewarded by teaching organisations and governments. The impact on future teaching efforts of an increasing number of practices not owned by GPs [25] is unknown and will need further monitoring and exploration.

Enjoyment of learning, a social aspect of learning, is important in sustaining learning and also in preventing burnout and teacher fatigue. Enjoyment has previously been identified as an important motivator for teachers [16] and has also been found in this research to be important for the learners. Finding ways of maintaining the enjoyment factor in learning will be important, particularly with the likely stresses of increasing clinical workload associated with an aging population and retiring GP workforce [26].

Responsiveness to the learners’ needs has long been recognised as important in effective learning [8]. The multi-level learning experience in community practice appears to meet the needs of individual learners.

#### Teaching infrastructure

Appropriately resourced teaching rooms within the general practice are essential for the expanded teaching effort. Government infrastructure grants for practices [27] as well as private investment from GP practice owners themselves have been important in providing additional consulting rooms and meeting rooms for learners [21]. Further infrastructure investment will be needed if the number of integrated practices is to increase.

Several of the integrated practices participating in this research had obtained government grants to extend their premises for the purposes of teaching. This had clearly been a prerequisite in enabling these practices to provide sufficient patient consulting space for multiple learners. Other practices had made their own investment to increasing their practice size, but indicated that they would need to evaluate the return on that investment.

Governments and funders will need to investigate the financial cost of models of integrated teaching and learning described in this report to ensure that general practices are being appropriately compensated for their teaching endeavours and for their innovation.

### Innovative teaching methods

GP supervisors in our research were responsive to needs of different level of learners and able to be flexible about the type of learning opportunities provided. New models of teaching and learning, including the supernumerary GP supervisor (“consultant GP teacher”), teaching teams and multi-level learner tutorials have been devised independently by GP supervisors in response to the needs of multi-level learners in the clinical general practice context. These supervisors are innovators in the clinical GP education environment and it is essential that teaching organisations learn from and support them to share their knowledge and expertise. New methods of learner centred teaching delivery need evaluation and appropriate funding [28].

The emerging supernumerary GP supervisor or GP consultant teacher model in some ways replicates the practice of specialist consultants in a teaching role in the public hospital environment. In this model, the consultant has a team of learners and junior staff working under his/her supervision and the consultant spends a significant time teaching. Remuneration and support for this emerging GP role needs exploring by teaching organisations and funders. This is a challenge in the Australian context due to the complex arrangements for funding teaching.

Other studies have found that the participation of all the practice staff, both administrative and clinical, is an important feature of successful learning [29]. Some practices had appointed a non-medical learning coordinator to support the teaching team, although these roles are not yet developed or funded. The increasing role of GP Registrars in these teaching teams is now being acknowledged [30]. This research confirmed that junior learners recognised that near – peer teaching provided specific benefits in the structure and content of the teaching, however, also confirmed some limitations in flexibility and clinical experience in near-peer teaching [31]. Participation by GP Registrars in teaching was not universal and the teaching arrangements were determined by the practice. Teaching organisations must recognise the unique contribution and skill sets of members of teaching teams and train and resource appropriately.

Multi-level learner group tutorials are valuable additions to the usual suite of learning opportunities for medical students, junior doctors, GP registrars and the GP supervisor in integrated practices. Training and support should be provided to GP supervisors and others involved in these tutorials to ensure quality tutorial delivery and to promote introduction of this form of learning in other teaching practices.

### Other models of teaching and learning

Some other learning methods were consistently valued highly by the learners. For example learners found parallel consulting very beneficial because they put learning into action [32]. This method of learning is becoming more main stream and needs to be further developed and introduced to new GP supervisors.

Informal teaching and learning exchange with learners at different levels is also seen as valuable. The capacity to meet with the team of clinicians in the tearoom in a general practice was referred to by many learners as providing valuable learning opportunity. This form of learning is part of the medical work based apprentice model of learning [33] and needs further exploration and research in context of multi-level learning.

Small rural hospitals also provided an opportunity for multi-level learning and this is perhaps an underutilised learning context that could be further developed.

### Limitations of this study

Difficulty in the recruitment of medical student participants was a significant limitation of this study. Teaching timetables, exam periods, university holidays and, in urban practices, the limited number of medical students placed in the practices of interest throughout the study’s duration all impacted ability to recruit medical students. The researchers were able to access only two students placed in urban practices. However, the experiences of rural stream medical students were sufficiently broad and correlated with data from the urban medical student interviews.

The study was based in one teaching region. In this region, the medical school and regional GP training provider have worked in an integrated manner to provide general practice training. At the time of the research, the medical school general practice academic unit delivered training to medical students in their GP term, junior PGPPP doctors and GP registrars. The academic unit arranged placements in general practices, delivered structured teaching sessions and trained GP supervisors for all levels of learner. These arrangements are unique in the Australian context. Therefore the findings of this study may not be immediately generalisable to other regions where administrative integration at a high level is not established.

Only eight general practices were selected to participate in this study. These practices were selected because they had experience delivering fully integrated learning to all four levels of learner. The findings may not reflect teaching in all teaching practices in this region. However, the aims of this research were to explore VI in a setting in which it was fully established, therefore the selection of these practices was integral to the research objectives. It would be interesting to explore the differences in learning experiences between VI practices and non-VI practices.

## Conclusion

We found vertical integration of GP education and training generally impacted positively on all levels of learner. VI learning enhances the learning environment as well as providing teaching structures and processes that have a positive impact on learners in the general practice setting.

Our findings provide insight into issues for educational organisations to consider in promoting and supporting VI teaching in general practices. Engaging with practices not owned by GPs may be a future challenge in delivering VI models training. It is evident that the practice environment is an important consideration in VI teaching and learning. Education organisations have a role in providing ongoing GP supervisor and other practice staff training for VI learning and support to teaching practices in securing appropriate teaching environments (e.g. sufficient practice consulting space).

Some of the more recent innovations in VI delivery such as the supernumerary GP supervisor (the consultant GP teacher), multi-level practice based group tutorials and the practice teaching team deserve further exploration and support. The important impact of informal learning in the “tearoom discussions” is another area for further evaluation and research.

## Endnote

^1^Parallel consulting (also known as wave consulting) is a common form of supervision used in these integrated practices [32]. It consists of a GP consultant seeing patients in parallel with either a medical student or PGPPP junior doctor (or at times both). The student or PGPPP junior doctor consults with patients in their own room, being allocated patients on a half hourly or hourly basis depending on experience level. Meanwhile, the GP supervisor sees his or her own patients. The GP supervisor has a lesser patient load than usual (often two patients an hour) to allow time to join the end of the junior learner’s consultation. The junior learner presents the patient to the GP supervisor and final decisions regarding patient management and follow up are made jointly.

## Abbreviations

GP: General practice; VI: Vertical integration of general practice education and training; PGPPP: Prevocational general practice placement program.

## Competing interests

The authors declare they have no competing interests.

## Authors’ contributions

All authors contributed to the concept and design of the study. JT and EH conducted the acquisition of data. JT, EH, KA, and AB contributed to the analysis and interpretation of data. JT drafted the manuscript and all authors participated in critical revision of the article and approved the final article.

## Pre-publication history

The pre-publication history for this paper can be accessed here:

http://www.biomedcentral.com/1472-6920/14/55/prepub
